# Impact of body mass index on outcomes of cardiac rehabilitation: a systematic review and meta-analysis

**DOI:** 10.3389/fcvm.2026.1757861

**Published:** 2026-05-22

**Authors:** Siwei Tu, Chunna Ding

**Affiliations:** Rehabilitation Medicine Department, Affiliated Hospital of Shaoxing University, Shaoxing, Zhejiang, China

**Keywords:** body fat, cardiorespiratory fitness, metabolic equivalents, obesity, overweight

## Abstract

**Objective:**

The effectiveness of cardiac rehabilitation (CR) after cardiac events and surgeries is well known. However, whether baseline body mass index (BMI) impacts CR outcomes is unclear. This first systematic review and meta-analysis of literature examined the difference in CR outcomes based on various BMI categories.

**Methods:**

The Web of Science, Embase, PubMed, and Scopus databases were searched until October 6, 2025, for all studies examining the outcomes of CR based on BMI. The primary outcome was the change in exercise capacity [as measured by changes in metabolic equivalents (METs)]. In contrast, secondary outcomes included changes in BMI, lipid profile, resting heart rate, and resting blood pressure.

**Results:**

Eighteen studies were included. In the comparison of obese vs. non-obese participants, pooled results showed no significant difference in the change in METs between the two groups. Subgroup analyses based on study design and obesity definition did not alter the overall findings. Among secondary outcomes, the change in BMI was not significantly different between the groups. Additionally, total cholesterol levels improved slightly more in non-obese individuals; however, there were no significant differences observed for high-density lipoprotein (HDL), triglycerides, heart rate, or blood pressure. In the comparison of overweight vs. normal BMI groups, no significant differences were observed for change in METs or BMI. Similarly, total cholesterol and HDL levels did not differ significantly between groups, although triglyceride reduction was greater among patients with a normal BMI. High heterogeneity was noted in most analyses.

**Conclusions:**

Our results indicate that pre-rehabilitation BMI does not affect improvements in exercise capacity and lipid profile in CR patients. Variations in CR protocols and high inter-study heterogeneity prohibit firm conclusions.

**Systematic Review Registration:**

https://www.crd.york.ac.uk/PROSPERO/view/CRD420251153489, identifier CRD420251153489.

## Introduction

Cardiac rehabilitation (CR) is a vital component of contemporary heart care. It provides a well-researched, team-based approach to help patients regain their strength and improve their long-term health after adverse cardiac events or surgeries (1). While protocols of CR vary widely in the literature, the program is often a combination of structured exercise, education, and behaviour changes. The common goal of all CR programs is to reduce adverse cardiovascular risk factors, improve both physical and mental health, and promote lasting lifestyle changes that lower the chances of future health issues (2, 3). Nevertheless, outcomes of CR are not consistent, and even with its known benefits, patients respond differently to CR (4).

Recent research has focused on body composition, especially body mass index (BMI), as a modifying factor on the effectiveness of CR (5, 6). Obesity, often identified by a BMI of ≥30 kg/m^2^, is a major global health problem linked to negative general and especially cardiovascular health. Excess body fat is frequently linked with inflammation and increased oxidative stress, leading to metabolic disease and alteration in the structure and functional capacity of the heart (7, 8). Additionally, people with higher BMIs often face challenges with physical activity and CR programs. Obesity related issues such as joint pain and reduced exercise ability can hinder the participation of high BMI patients in rehabilitation programs (9).

On the other hand, CR presents a key chance for individuals with high BMIs to change their lifestyles and improve their heart fitness, even if they have obesity-related health problems. The organized exercise and nutritional support offered in CR programs can help individuals lose weight and improve metabolic health (10). However, it is still unclear how effective CR is for this group. Some studies suggest that responses to exercise, quality of life improvements, and cardiorespiratory fitness changes might differ across various BMI levels (6, 11, 12).

Understanding the link between BMI and CR outcomes is vital for enhancing patient care, particularly in reducing the chances of disease recurrence and improving heart health. Figuring out how BMI affects functional and metabolic to CR could aid in developing more tailored, inclusive, and effective rehabilitation programs. Although several studies (6, 11, 12) have looked into how BMI influences CR, there is still no comprehensive review that gathers these findings to provide solid evidence. Thus, the primary aim of this systematic review and meta-analysis was to quantitatively evaluate the impact of baseline BMI on functional and cardiometabolic outcomes following CR, with particular emphasis on change in exercise capacity measured by metabolic equivalents (METs). Secondary aims were to compare changes in BMI, lipid parameters [total cholesterol, high-density lipoprotein (HDL), triglycerides], heart rate, and blood pressure between obese and non-obese individuals undergoing CR.

## Material and methods

PRISMA guidelines (13) were followed during the conduct of this systematic review. This systematic review and meta-analysis were prospectively registered in the PROSPERO database (CRD420251153489).

### Inclusion and exclusion of studies

This review used the PECOS framework to establish the inclusion and exclusion criteria. P: Population of adult patients undergoing CR after any cardiac event or cardiac procedure/ surgery. E & C: *Exposure* consisting of patients with high BMI *Compared* to patents with low BMI. BMI categories were not pre-defined and all BMI-based obesity definitions (Western or Asian) were eligible O: Outcome consisting of any of the following: exercise capacity or tolerance, lipid profile or cardiovascular adverse events. As for S, all study designs were eligible.

Exclusion criteria were: Non-comparative studies, unpublished data, not segregating data based on BMI, and those with overlapping data.

### Search strategy and study selection

Two independent reviewers performed the literature search and evaluated all articles for eligibility. Discrepancies among the reviewers at any stage were reconciled through deliberations. A thorough literature search was performed in the Web of Science, Embase, PubMed, and Scopus databases for all pertinent papers, without language restrictions. The search phrases included both free-text and Medical Subject Headings. A comprehensive search approach is available in Supplementary Table S1. The search concluded on October 6, 2025.

The selection of research was based on a comprehensive analysis of the search results, which included preliminary deduplication, followed by screening of the titles and abstracts of the studies. The reviewers assessed the study titles and abstracts according to the eligibility criteria to choose articles for additional assessment. Selected studies were subjected to full-text review, and inclusion in this review occurred upon consensus between both reviewers.

### Data management

The reviewers prepared a data extraction form consisting of the following subheadings to extract relevant information: study details, location, design, demographics, BMI-based groups, comorbidities, CR protocol, outcomes and results. Outcome data were extracted for all results reported by the studies. All results were descriptively presented while similar outcomes were quantitatively analyzed. Study authors were not contacted for missing data. Data from the studies was segregated for two primary comparisons: Obese vs. non-obese and overweight vs. normal BMI. For the non-obese group in the first comparison, data of overweight and normal BMI groups was merged to formulate a single “non-obese” group. The primary outcome was change in exercise capacity (change in METs) while secondary outcomes were change in BMI, lipid profile, resting heart rate, and resting blood pressure.

### Risk of bias

We used the Newcastle-Ottawa Scale (NOS) (14) to assess methodological quality and risk of bias in observational studies, which has been validated for use in case-control and cohort study designs. Each study was assigned a domain-specific star rating (0–9 total), with higher scores suggesting reduced risk of bias (≥seven stars = low risk; 4–6 = moderate; ≤3 = high). Two reviewers conducted separate assessments, and discrepancies were resolved through conversation.

### Data analysis

All statistical analyses were performed using random-effects meta-analyses (DerSimonian–Laird method) to account for potential between-study heterogeneity. For each study, mean change (Δ) in outcomes was calculated as “Post-intervention mean minus Pre-intervention mean (Δ = Post − Pre)”. The corresponding standard deviation (SD) of change was estimated using the formula “SD(*Δ*) = √[SD₍post₎^2^ + SD₍pre₎^2^ − 2r·SD₍post₎·SD₍pre₎]”, assuming a pre–post correlation (r) of 0.7 when not reported. For each outcome, Hedges' g was calculated as the standardized mean difference between groups (obese vs. non-obese or overweight vs. normal BMI) using pooled SD. 95% confidence intervals (CIs) and *p*-values were computed for pooled effect sizes, and heterogeneity across studies was quantified using the Cochran's Q statistic and the *I*^2^ statistic, with *I*^2^ values of 25%, 50%, and 75% interpreted as low, moderate, and high heterogeneity, respectively. Meta-analyses and all supporting computations were conducted using “R” software with the metafor, readxl, officer and flextable packages. Sensitivity analyses were performed for the primary outcome using a leave-one-out approach, where each study was sequentially excluded to assess the influence of individual studies on the pooled effect estimate and heterogeneity. Additionally, subgroup analyses were conducted to explore potential sources of heterogeneity based on study design (retrospective vs. prospective) and obesity definition (BMI ≥30 kg/m^2^ vs. lower thresholds). A funnel plot and Egger's regression test were also used to assess publication bias for the primary outcome. Statistical significance was set at *p* < 0.05 for all analyses.

## Results

### Search results

**Figure 1:**
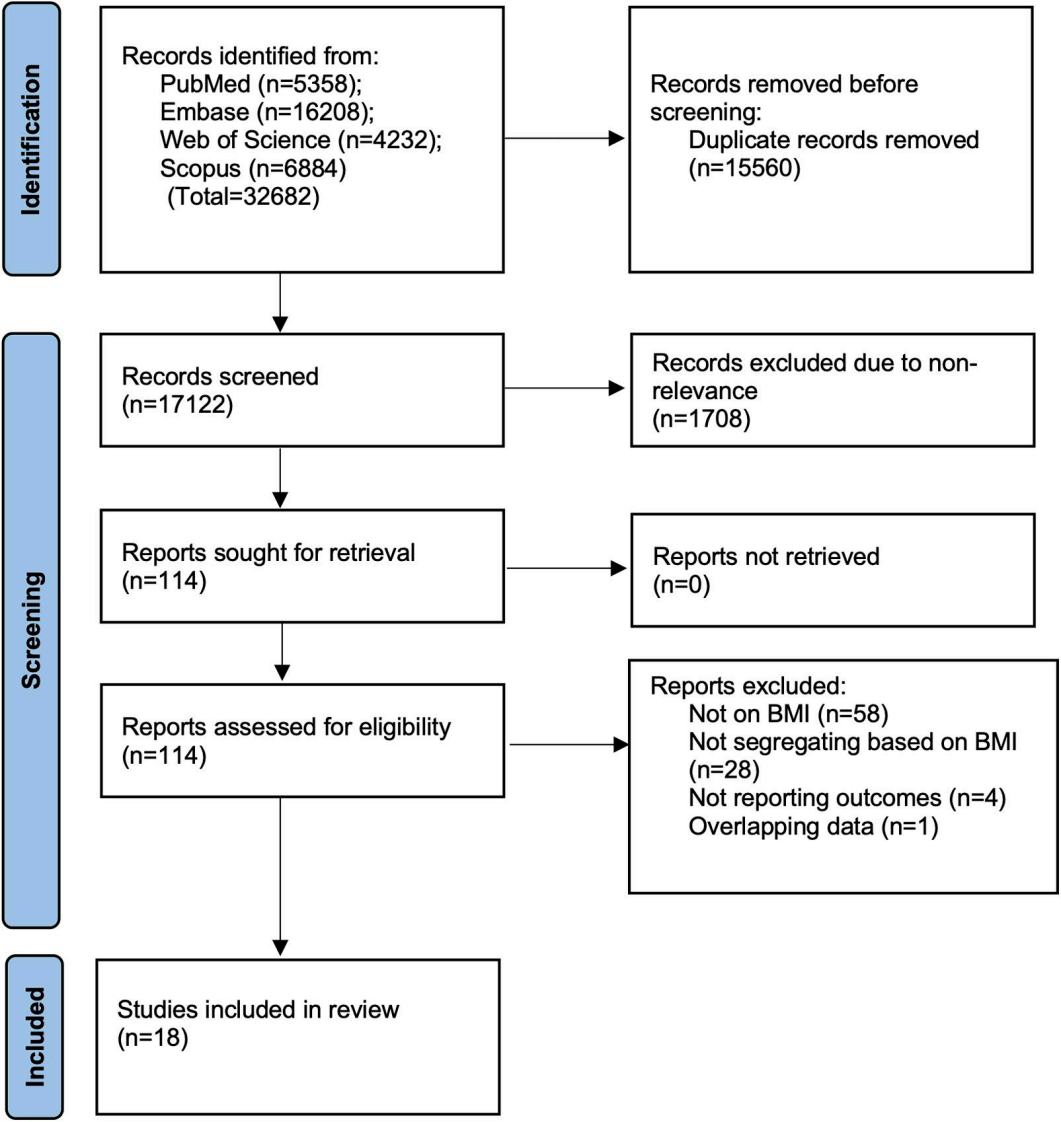
PRISMA flowchart.

A total of 32,682 records were found through electronic database searches (Figure 1). After removing 15,560 duplicate records, 17,122 titles and abstracts were reviewed for relevance. Of these, 17,008 records were excluded because they were not relevant, leaving 114 full-text articles for detailed evaluation. After reviewing the full texts, 96 reports were excluded for various reasons. Ultimately, 18 studies (5, 6, 11, 12, 15–28) met the inclusion criteria and were included in the systematic review.

### Study details and outcomes

The baseline characteristics of the included studies are summarized in Table 1. The studies were published between 1996 and 2024, with a total pooled sample of over 12,000 participants who underwent structured cardiac rehabilitation (CR) programs. Most studies took place in North America (USA, Canada), with additional contributions from Europe, the Middle East, and Asia. Most studies used a retrospective cohort design, while six studies were prospective. Participants were mostly male (between 55% and 90%), with average ages in the mid-50s to late 60s. The primary cardiac diagnoses included coronary artery disease, acute coronary syndrome, and heart failure. Definitions of obesity varied slightly but were generally consistent with BMI of 30 kg/m^2^ or higher, while studies conducted in Asian populations used lower thresholds, typically 25–27 kg/m^2^. CR programs were usually multicomponent outpatient programs, including aerobic and resistance exercise training, as well as education, nutrition counseling, and lifestyle change sessions. Program durations ranged from 4 weeks to 6 months, with most lasting between 8 and 12 weeks. All studies recorded baseline data at the start of the program while final outcome data was recorded at completion of the same.

**Table 1:** Details of included studies.

Author	Year	Location	Study design	Primary cardiac disease	Groups	Definition as per BMI (kg/m^2^)	Sample size	Age (years)	Males (%)	DM (%)	HT (%)	CR program details
Lavie et al.	1996	USA	RC	CAD	Obese	≥27.3–27.8	116	58	NR	27	71	3-month structured outpatient program, 36 supervised sessions (3/week). Each session included aerobic exercise (treadmill or cycle ergometer) tailored to target HR, plus education, dietary counseling, and behavioral modification.
					Non-obese	<27.3–27.8	198	62		22	60	
Bader et al.	2001	USA	RC	CAD	Obese	≥30	240	56	62.3	29.6	57.1	10–12 week outpatient phase II CR. Participants attended supervised exercise sessions 2–3×/week involving aerobic activities (walking, treadmill, cycling) at 60%–85% of HR max. Also included health education and counseling on diet, medication, and stress management. No special weight loss intervention.
					Overweight	25–29.9	157	61	75	17.8	44	
					Normal	<25	52	56	50	11.5	34.6	
Shubair et al.	2004	Canada	RC	CAD	Obese	≥30	1,230	60	73.9	33.9	63.1	24-week comprehensive CR. Structured exercise sessions focusing on aerobic endurance (treadmill, stationary bike), strength, and flexibility training. Frequency 2–3×/week under supervision. Education on risk factor modification, nutrition, smoking cessation, and stress control included.
					Overweight	25–29.9	1,648	63.9	82.7	21.1	53.8	
					Normal	<25	644	65.8	74.1	16.5	51.7	
Sierra-Johnson et al.	2005	USA	PC	CAD	Obese	≥30	114	59	71.1	19	62	CR of 4–8 weeks duration. 1–3 supervised sessions per week, involving treadmill and cycling aerobic exercises individualized to each patient. Participants received lectures on exercise, medications, and dietary modification, with at-home exercise encouraged.
					Overweight	25–29.9	195	62	88.2	15	52	
					Normal	18–24.9	80	63	68.8	16	48	
Gunstad J et al.	2007	USA	RC	CAD	Obese	≥30	149	63.4	94	6.6	14.6	Standard phase II CR focusing on supervised aerobic and resistance training. Sessions typically 3/week, 1 h each. Exercise included treadmill, cycle ergometer, and resistance exercises. Program emphasized improving cardiorespiratory fitness and psychosocial well-being.
					Overweight	25–29.9	173	66.8	87	12	28	
					Normal	18.5–24.9	71	70	95	4	11	
Ghashghaei et al.	2012	Iran	PC	CAD	Obese	≥30	84	NR	0	NR	NR	2-month comprehensive CR program (24 sessions; 3/week). Each session lasted ∼90 min (20 min warm-up, 60 min aerobic/resistance, 10 min cool-down). Activities included treadmill, cycle ergometer, stair climbing, and rowing. Intensity 60%–85% max HR. Nutritional, psychological, and educational sessions included.
					Non-obese	<30	121		0			
Martin et al.	2012	Canada	RC	CAD	Obese	≥30	993	57.8	79.4	23.0	66.5	12-week supervised CR (2×/week supervised + 2–3×/week home). Aerobic, resistance, and stretching training; intensity individualized by treadmill testing; target HR guided. Included dietary, psychological, and educational counseling.
					Overweight	25–29.9	1,929	60.4	81.2	11.9	56.2	
					Normal	18.5–24.9	1,075	61.4	72.7	12.7	48.8	
Xu et al.	2015	China	PC	ACS	Obese	≥25	29	48.8	96.3	33.3	70.4	7-week CR (2–3-week inpatient + 4–5-week outpatient). Inpatient: education, counseling, risk management, nutrition, postoperative mobility. Outpatient: 3×/week (5-min warm-up, 20-min aerobic walking/trotting, 10-min cool-down) at 60% HR max (Borg 11–12).
					Normal	<25	32	50.4	88.2	38.2	26.5	
Lim et al.	2016	Korea	RC	AMI	Obese	≥25	170	54.3	86.5	22.4	41.2	Hospital-based or home-based phase II CR. Exercise prescription based on treadmill test and modified Bruce protocol. Patients advised to exercise ≥3/week at target HR (60%–85% HRmax). Supervised sessions in hospital and home programs monitored via diary. Program duration 4–8 weeks.
					Normal	<25	189	59.1	78.8	21.7	40.2	
Pieters et al.	2018	Nether-lands	RC	CAD	Obese	≥30	45	58	89	22	53	12-week, 2×/week (1.5 h) group CR program: aerobic, resistance, and flexibility training; supervised by multidisciplinary team; education and lifestyle counseling (nutrition, psychology, smoking cessation).
					Overweight	25–29.9	122	59	88	12	34	
					Normal	18.5–24.9	75	58	69	8	35	
Braga et al.	2019	Portugal	RC	ACS	Obese	≥30	167	53.4	81	26	56	CR was in two phases. Phase I, during hospital stay, included educational programs for patients about
					Non-obese	<30	564	54.5	88	16	37	their disease and control of CV risk factors. Early mobility was also encouraged. Phase II consisted of an outpatient multi-professional intervention, which started 2 to 3 week after hospital discharge. This phase included supervised exercise training, nutritional assessment, as well as smoking cessation counseling.
Terada et al.	2019	Canada	RC	All CVD	Obese	≥30	201	62	75	31	85	Patients participated in 1 of 3 CR programs: on-site, home-based, or brief. All 3 CR programs included on-site coronary risk factor management consisting of a risk-factor modification consultation with a physiotherapist or registered nurse. The on-site exercise program consisted of 60 min supervised exercise training sessions twice weekly for up to 3 months
					Overweight	25–29.9	264	62	77.3	15.2	82.6	
					Normal	18.5–24.9	117	65	67.5	11.1	79.5	
Atti et al.	2021	USA	RC	CAD	Obese	≥30	91	64.4	70.3	46.2	78	12-week outpatient CR with supervised aerobic and resistance training, 3×/week at 60%–85% HR max.
					Non-obese	<30	87	66.4	71.3	31	81.6	
El Missiri et al.	2021	Egypt	PC	CAD	Obese	≥30	62	52	80	38.7	54.8	12-week CR including aerobic and resistance training, 3×/week for 45–60 min.
					Non-obese	<30	58	54.2	94	24.1	27.6	
Khan et al.	2021	USA	RC	HF	Obese	≥30	34	58.2	62	62	85	CR (36 sessions, 3×/week) with aerobic + resistance exercise.
					Non-obese	<30	74	61.2	78	30	53	
Peters et al.	2022	Nether-lands	PC	HF	Obese	≥30	105	713.	48.6	68.6	95.2	Multicomponent CR over 6 months (2×/week). Included aerobic, balance, and resistance training.
					Non-obese	<30	70	75.7	55.7	48.3	84.3	
Conradson et al.	2024	UK	RC	CAD	Obese	≥30	398	59	0	29.6	72.6	12-week CR (2×/week, supervised aerobic + resistance training)
					Overweight	25–29.9	461	64	0	17.6	64	
					Normal	18.5–24.9	454	63	0	12.1	55.1	
Mittal et al.	2024	USA	RC	All CVD	Obese	≥30	75	64	71	NR	NR	Standard and intensive CR (36–72 h) with aerobic treadmill training
					Non-obese	<30	226	67	69			

RC, retrospective cohort; PC, prospective cohort; CAD, coronary artery disease; CVD, cardiovascular disease; BMI, body mass index; CR, cardiac rehabilitation; HR, heart rate; AMI, acute myocardial infarction; ACS, acute coronary syndrome; HF, heart failure.

A comprehensive enumeration of outcomes and results from all included studies is provided in Table 2. The 18 studies investigated various clinical and functional outcomes to evaluate the effect of obesity on CR response. Nearly every study examined exercise capacity—typically quantified as peak METs—as a primary endpoint, in conjunction with cardiometabolic indicators such as lipid profile, modifications in BMI, and hemodynamic parameters. Notwithstanding lower baseline fitness levels, obese patients typically exhibited substantial enhancements in functional capacity post-CR, with the majority of studies indicating similar increases in peak METs compared to normal-BMI individuals.

**Table 2:** Summary of outcomes and results of included studies.

Author	Outcomes Assessed	Results
Lavie et al.	Exercise capacity (peak METs), weight, lipid profile, and blood pressure changes after CR.	Obese patients had lower baseline fitness but achieved similar improvements in exercise capacity (METs) and lipid risk factors after 3 months of CR. Both obese and non-obese groups showed significant improvement in functional capacity.
Bader et al.	Exercise tolerance (treadmill time, METs), lipid profile, and weight change after 12-week CR.	All BMI groups improved in exercise tolerance and peak METs. Obese patients showed smaller reductions in weight and lipids but similar gains in exercise capacity.
Shubair et al.	Exercise capacity (peak METs), metabolic profile (lipids, glucose), and functional outcomes after CR.	Obese and overweight patients had lower baseline METs but demonstrated significant improvements in exercise capacity and lipid parameters after CR, comparable to normal-weight participants.
Sierra-Johnson et al.	Long-term cardiovascular mortality and morbidity after CR participation.	Obese patients had similar or lower cardiovascular mortality compared with normal-weight participants over long-term follow-up, supporting an “obesity paradox” in CR outcomes.
Gunstad J et al.	Functional work capacity (METs) and quality of life (SF-36 physical and mental scores).	All BMI groups improved in METs and quality of life after CR. Obese patients showed significant gains in both physical and mental components, with comparable improvements to non-obese participants.
Ghashghaei et al.	Functional capacity (METS), lipid profile, fasting blood sugar, and BMI changes after 2-month CR.	Both obese and non-obese women showed significant improvement in METs and lipid profile. BMI and triglycerides decreased significantly post-CR in both groups, with similar magnitude of improvement.
Martin et al.	Aerobic capacity (peak METs) improvement after CR and at 1-year follow-up.	All BMI groups improved post-CR. However, obese patients had smaller short-term and 1-year improvements in METs compared with non-obese patients, suggesting limited long-term retention of CR benefits in obesity.
Xu et al.	Exercise capacity (6MWT, METs), echocardiographic parameters (LV function, ejection fraction), and BMI change after CR.	Both normal and overweight/obese groups improved significantly in LV function and METs after CR. Obese patients showed better improvement in functional parameters but less change in BMI.
Lim et al.	Cardiopulmonary exercise capacity (peak METs, VO2max), resting heart rate, and blood pressure changes after CR.	Both obese and non-obese patients improved in peak METs and exercise duration. No significant between-group differences were seen, though obese patients showed greater relative improvement in METs.
Pieters et al.	Self-reported physical and mental health, exercise capacity, and BMI changes after CR.	All BMI categories showed significant improvements in physical and mental health scores after 12-week CR. Obese patients benefited similarly to normal and overweight patients in subjective recovery and exercise outcomes.
Braga et al.	Exercise capacity (METs), chronotropic index, resting heart rate RHR, and weight change after CR.	Obese patients had lower baseline exercise capacity and chronotropic index but achieved greater relative improvement in exercise capacity after CR. CI improved significantly in both groups, while resting heart rate reduction was not significant.
Terada et al.	Cardiorespiratory fitness (peak VO₂, METs), body composition (fat mass, lean mass), and blood pressure after exercise-based CR.	Both obese and non-obese groups improved cardiorespiratory fitness and reduced fat mass following CR. Improvements in VO₂ peak were similar across BMI groups, indicating comparable CR benefits regardless of obesity status.
Atti et al.	Chronotropic competence, blood pressure response, and METs pre- and post-CR.	Both obese and non-obese patients demonstrated significant improvement in chronotropic competence and METs after CR. Improvement in blood pressure response was significant only among non-obese patients. CR benefits occurred irrespective of weight loss.
El Missiri et al.	BMI, blood pressure, lipid profile, fasting glucose, LVEF, and exercise capacity (METs) after 12-week CR.	Obese patients had greater reductions in BMI, blood pressure, and LDL-C compared with non-obese. Both groups improved exercise capacity, but only non-obese showed a greater increase in LVEF.
Khan et al.	Cardiorespiratory fitness (peak METs), hospitalization, and all-cause mortality in HF patients after phase II CR.	Obese patients had lower baseline and post-CR fitness and smaller percentage improvement in METs. There were no differences in 5-year mortality or 2-year hospitalization rates between BMI groups.
Peters et al.	Physical function, 6MWD, and quality of life (KCCQ) at 3 and 6 months post-CR in acute HF patients.	Older patients with and without obesity benefited from rehabilitation therapy. Improvements in physical function were larger among obese patients, particularly in the balance component, while 6MWD and KCCQ improvements were greater among non-obese patients.
Conradson et al.	Cardiorespiratory fitness (peak METs), blood pressure, lipids, and waist circumference pre- and post-CR in women by BMI category.	Women with normal and overweight BMI achieved significant cardiorespiratory fitness improvements, while those with obesity showed smaller gains. All groups improved in lipids and waist circumference, but obese women showed less fitness improvement.
Mittal et al.	Cardiorespiratory fitness (METs) and weight change after CR participation.	Both obese and non-obese patients significantly improved METs. Weight loss was greater in obese patients. CR improved fitness in all groups, though gains were smaller in obesity.

METs, metabolic equivalents; CR, cardiac rehabilitation; BMI, body mass index; 6MWD, 6-minute walk distance; HF, heart failure; LVEF, left ventricular ejection fraction;.

A few of the studies did not provide coherent data for a quantitative analysis and hence were assessed only descriptively. Sierra-Johnson et al. examined only long-term cardiovascular mortality and nonfatal recurrent events and found that obesity was inversely related to cardiovascular mortality but directly related to nonfatal recurrent events. Pieters et al. (22) examined only subjective health status based on the Short Form-12 questionnaire between different BMI groups and noted that overweight patients had the best improvement while there was no statistical significant difference between obese and normal BMI patients in the outcomes. Terada et al. (20) compared various cardiorespiratory fitness indicators, body composition and hemodynamic parameters between various BMI groups but did not report numerical values for a meta-analysis. The authors showed that improvements were similar across BMI categories. Khan et al. (23) showed that obese patients had lower improvement in MET but no difference in 5-year mortality or 2-year hospitalization rates as compared to normal BMI group. Peters et al. (27) compared obese vs. non-obese patients undergoing CR with Short Physical Performance Battery, 6-minute walk distance, and the Kansas City Cardiomyopathy Questionnaire and noted that outcomes did not differ between BMI categories.

### Obese vs. Non-obese

**Figure 2:**
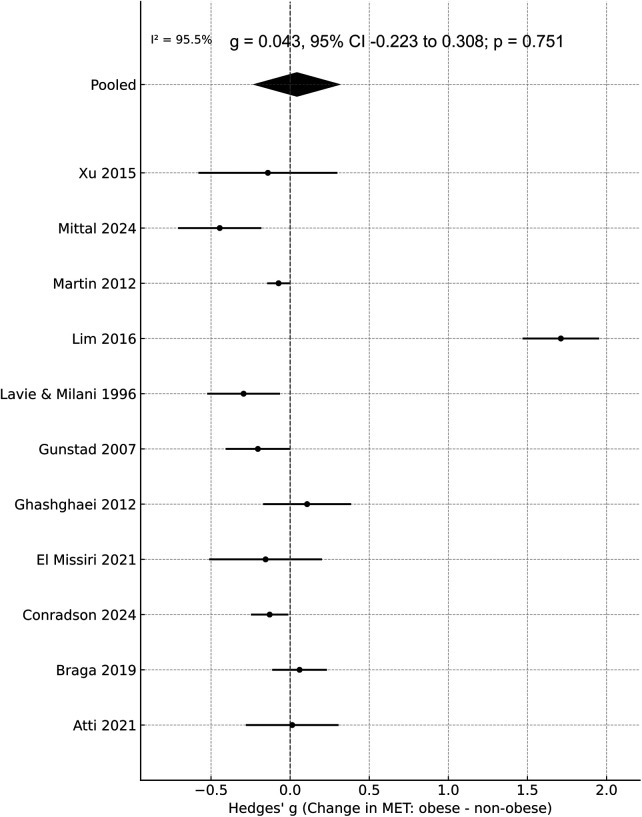
Meta-analysis of change in METs between obese vs. non-obese groups undergoing CR.

This comparison included eight key outcomes assessing the impact of obesity on CR response. For exercise capacity, the pooled effect on change in METs was small and not statistically significant (g = 0.043, 95% CI −0.223 to 0.308; *p* = 0.751), with considerable heterogeneity (*I*^2^ ≈ 96%) (Figure 2). There was no major asymmetry detected on funnel plot (Supplementary Figure S1). The Egger's test was non-significant (*p* > 0.05), indicating no evidence of publication bias. However, on sensitivity analysis, the exclusion of the study of Lim et al. (25) indicated a statistically significant improvement in non-obese group as compared to obese group g = −0.116, 95% CI −0.202, −0.030, *I*^2^ ≈ 48%) (Supplementary Table S2). Subgroup analysis results are shown in Table 3. The results did show change in the significance of the summary estimates when segregated based on design. But on subgroup analysis based on obesity definition, it was noted that obese patients had significantly reduced improvement in METs when defined as ≥30 kg/m^2^.

**Table 3:** Subgroup meta-analysis results for change in MET.

Variable	Group	Studies	Hedges' *g*	95% CI	I^2^ (%)
Study design	Retrospective	8	0.077	(−0.248, 0.402)	96.8
	Prospective	3	−0.022	(−0.217, 0.174)	0.0
Obesity definition	≥30 kg/m^2^	8	−0.097	(−0.189, −0.005)	51.0
	other	3	0.429	(−0.993, 1.852)	98.7

CI, confidence intervals.

BMI change showed no significant difference between obese vs. non-obese individuals (g = –0.23, 95% CI −0.48 to 0.02, *p* = 0.07, *I*^2^ = 91%) (Supplementary Figure S2). Among lipid outcomes, a significant pooled effect was observed for total cholesterol favoring greater improvement in the non-obese group (g = –0.10, 95% CI −0.16 to −0.04, *p* < 0.001, *I*^2^ = 0%) (Supplementary Figure S3), while changes in HDL (g = –0.02, 95% CI −0.17 to 0.14, *p* = 0.83, *I*^2^ = 80%) (Supplementary Figure S4) and triglycerides (g = –0.04, 95% CI −0.16 to 0.08, *p* = 0.49, *I*^2^ = 71%) (Supplementary Figure S5) were not significant. No significant differences were found for hemodynamic measures, including heart rate (g = –0.01, 95% CI −0.12 to 0.11, *p* = 0.90, *I*^2^ = 0%) (Supplementary Figure S6), systolic blood pressure (g = –0.09, 95% CI −0.20 to 0.02, *p* = 0.12, *I*^2^ = 0%) (Supplementary Figure S7), or diastolic blood pressure (g = 0.01, 95% CI −0.10 to 0.12, *p* = 0.84, *I*^2^ = 0%) (Supplementary Figure S8).

### Overweight vs. normal BMI

The pooled estimates for the comparison of overweight vs. normal BMI are summarized in Table 4. Briefly, for METs the pooled Hedges' g was −0.035 (95% CI −0.095 to 0.026), *p* = 0.25, *I*^2^ = 0.0%, indicating no significant difference in change in exercise capacity between the two groups. The meta-analysis also showed (g = −0.109, 95% CI −0.233 to 0.015, *p* = 0.08, *I*^2^ = 36.6%), no significant difference in BMI reduction between the groups. Likewise, change in total cholesterol (g = −0.077, 95% CI −0.164 to 0.010, *p* = 0.08, *I*^2^ = 0.0%) and (g = −0.052, 95% CI −0.237 to 0.133, *p* = 0.58, *I*^2^ = 77.7%) also demonstrated a non-significant difference. Finally, for triglycerides, the meta-analysis (g = −0.146, 95% CI −0.232 to −0.059, *p* = 0.001, *I*^2^ = 18.7%) showed a small but statistically significant greater reduction in triglycerides in the normal BMI group.

**Table 4:** Meta-analysis results: overweight vs. normal BMI.

Outcome	Number of Studies	Hedges' g	95% CI	*p*-value	*I*^2^ (%)
MET	4	−0.035	(−0.095, 0.026)	0.25	0.0
BMI	2	−0.109	(−0.233, 0.015)	0.08	36.6
Total cholesterol	2	−0.077	(−0.164, 0.010)	0.08	0.0
HDL	3	−0.052	(−0.237, 0.133)	0.58	77.7
Triglycerides	3	−0.146	(−0.232, −0.059)	0.001	18.7

CI, confidence intervals; HDL, high-density lipoprotein; MET, metabolic equivalents; BMI, body mass index.

### Risk of bias

Quality assessment of included studies is shown in Supplementary Table S3. None of the studies ensured comparability of various BMI groups and hence were not awarded any points for the same. Overall, the scores ranged from six to seven.

## Discussion

The present meta-analysis is the first in literature to provide collated evidence on the influence of obesity and overweight status on the outcomes of CR. Examining 18 studies from different countries and conducted over the past two decades, we noted that patients undergoing CR had significant improvements in exercise capacity and cardiometabolic parameters irrespective of the BMI categories. Importantly, the pooled results demonstrate that obesity does not diminish the beneficial effects of CR, as both obese and non-obese individuals achieved comparable gains in functional capacity and metabolic health following CR. When comparing overweight vs. normal BMI participants, the results were consistent with the obese vs. non-obese analysis. Improvements in METs, BMI, and lipid profile were comparable between groups, indicating that individuals with elevated BMI also respond equally well to CR programs.

Cardiorespiratory fitness is often acknowledged as a “vital sign” in heart care because to its significant prognostic capacity for long-term cardiovascular incidents and overall mortality in adults (9, 29). In women, a 1-MET enhancement in cardiorespiratory fitness correlates with a 17%–25% reduction in mortality risk, a benefit that seems proportionally more significant than in men, where a 1-MET increase is generally associated with a 12%–14% decrease in mortality risk (9). A large body of evidence confirms a continuous inverse correlation between cardiorespiratory fitness and mortality in both genders, with cardiorespiratory fitness demonstrating superior predictive strength compared to established risk scores (1, 30–32). Moreover, elevated fitness levels correlate with reduced rates of hospitalization and future cardiovascular incidents (1, 30–32). It is important to note that CR is not merely exercise training; rather, it is a multidisciplinary intervention that integrates risk-factor modification, medication optimization, nutrition and behavioral counseling, psychosocial support, and lifestyle education (2). Within this comprehensive framework, enhancements in cardiorespiratory fitness coincide with improvements in blood pressure, lipid profiles, endothelial function, and autonomic equilibrium (2). Nonetheless, enhancement in cardiorespiratory fitness is not observed in all individuals participating in CR. A 13-year retrospective investigation indicates that 22% of individuals participating in CR do not enhance their cardiorespiratory fitness, while 17% exhibit minimal gains (1). A study indicated that the mean baseline METs was the most significant predictor of cardiorespiratory fitness upon completion of CR and additional characteristics, such as sex, age, current smoking status, obesity, and diabetes, were strongly predictive of post-CR cardiorespiratory fitness (4).

The present investigation focused on one such factor, i.e., obesity, to provide high-quality evidence on this important variable for practicing clinicians as well as patients. Since baseline METs is one of the prime predictors of CR outcomes, we chose to use change in METs score for the meta-analysis rather than relying only on post-CR METs to elicit exact change recorded in various BMI groups. Descriptive analysis showed that most studies found that obese patients had similar gains in METs as compared to non-obese individuals entering CR. This was further validated by our quantitative analysis wherein we noted no statistical significant difference in change in METs between obese vs. non-obese and overweight vs. normal BMI patients at the end of the CR programs. The subgroup and sensitivity analysis more or less confirmed these findings as results remained stable across most subgroups and on sequential exclusion of majority studies. We did note better improvements in non-obese group as compared to obese group in studies using the cut-off of ≥30 kg/m^2^. It's important to recognise that statistical significance doesn't always equate to clinical significance. While some outcomes showed statistically significant differences between BMI groups, the effect sizes were consistently small, indicating these differences are unlikely to result in meaningful clinical benefits for individual patients. Therefore, caution is advised when interpreting these results.

Likewise, the meta-analysis also showed that changes in lipid profile were mostly similar across BMI subgroups. Indeed, total cholesterol showed significant improvement in the non-obese group while triglyceride levels demonstrated greater change in the normal BMI group, but all with very small effect sizes. The low number of studies in these meta-analyses as compared to the primary outcome could be one of the reasons for such results. Likewise, the number of studies reporting hemodynamic parameters were also scarce and while our results did not show any significant differences between obese and non-obese groups, the results must be interpreted with caution.

Another reason for a cautious interpretation of our results is the high inter-study heterogeneity noted in most of the meta-analysis. We believe that variations in the CR protocols is a major source of such heterogeneity along with contributions from differences in baseline patient characteristics, primary cardiac disease, BMI cut-offs and follow-up. The absence of standard definition renders the comparison of various CR programs exceedingly challenging. As seen in our review, the included studies differed markedly in program duration, frequency, exercise intensity, training modality, supervision, and adjunctive interventions. The setting and supervision levels also differed: some trials used fully supervised, center-based phase II programs, while others integrated or compared home-based and hybrid CR models. Variations in exercise prescription customization, whether derived from stress testing, symptom-limited protocols, or predetermined target heart rates, may have affected the extent of enhancement in METs. Many studies recommended aerobic exercise, whereas others employed variable amount of resistance training. Aerobic exercise often refers to low-intensity, steady-state exercises such as cycling or running. Resistance exercise generally refers to bodyweight activities (e.g., push-ups or yoga), the use of resistance bands, or isolated workouts targeting specific muscles. These represent distinct activity sets that impose varying degrees of stress on participants, and their relative efficacy in individuals with cardiovascular disease has not been comprehensively examined (33). Moreover, one must remember that effectiveness of CR requires high degree of compliance, a variable which was not consistently reported by the included studies. Additional challenges specific to the cardiovascular disease population include weariness, fear of another myocardial infarction, deconditioning, musculoskeletal pain, limited access to gyms, and unfamiliarity with exercise equipment (9). It was unclear from the studies how efficiently was the CR program conducted, a factor which could have contributed to the heterogeneity. Furthermore, population-level differences in baseline cardiovascular risk, disease severity, and healthcare infrastructure across countries (North America, Europe, Asia, and the Middle East) likely compounded the inter-study heterogeneity. Despite the high statistical heterogeneity, the quantitative synthesis was deemed appropriate because the studies were clinically similar. All focused on structured exercise-based CR and reported changes in similar outcomes. Although the effect sizes varied in magnitude, the overall direction of the effects was consistent, with both obese and non-obese participants showing comparable improvements in exercise capacity and cardiometabolic health. Using a random-effects model helped address the variability between studies, and subgroup and sensitivity analyses did not significantly change the main conclusions.

From a physiological standpoint, the lack of significant difference in exercise capacity and lipid profile noted between obese and non-obese individuals may be attributed to the preserved peripheral and muscle adaptations to training, irrespective of the presence of excess adiposity. Consistent aerobic and resistance training during CR helps enhances skeletal muscle oxidative capacity, mitochondrial efficiency, and endothelial function, resulting in improved oxygen supply and utilization irrespective of pre-rehabilitation BMI (10). Furthermore, persons with obesity frequently possess a higher absolute muscle mass, potentially enhancing submaximal strain tolerance and metabolic efficiency through training (34). Enhancements in cardiac output, stroke volume, and ventilatory efficiency have been documented post-CR in obese patients, suggesting similar functional improvements as compared to non-obese persons (10, 35). Therefore, the results of this systematic review indicate that the physiological response to structured CR may be independent of BMI, emphasizing the crucial importance of consistent physical activity and cardiovascular conditioning, rather than weight status alone, in facilitating significant enhancements in aerobic performance and clinical outcomes.

We must acknowledge the limitations of this review. Firstly, not all included studies were amenable for a meta-analysis primarily due to variations in data reporting. Not all included studies reported lipid profile and hemodynamic parameters which led to reduced number of studies in these meta-analyses. Secondly, the definition of obesity was also variable in the included studies which is in line with cut-offs used for Asian and Western populations (36). Thirdly, a significant limitation of this meta-analysis is that the majority of included studies employed a retrospective design and reported unadjusted or minimally adjusted outcomes, thereby limiting causal inference. In numerous studies, BMI groups were not balanced with respect to baseline cardiovascular risk factors, including age, sex distribution, comorbidity profiles, baseline cardiorespiratory fitness, and pharmacotherapy. Such imbalances may confound the observed relationships between BMI categories and changes in exercise capacity or cardiometabolic outcomes following CR. In the absence of consistently reported, fully adjusted effect estimates or individual patient-level data, the possibility of residual confounding cannot be dismissed. Fourthly, the review could not evaluate the association between BMI and training exercise intensity during CR. Although most included studies specified target intensity prescriptions, exercise intensity was not uniformly reported or stratified according to BMI category, and objective measures of achieved or adherence-adjusted training intensity were largely unavailable. Fifthly, the pre–post correlation coefficient (*r* = 0.7) was assumed to calculate the variance of change scores when this information was not reported by the original studies. While this value is widely applied in exercise and rehabilitation meta-analyses, the assumption may alter the precision of the estimated effect sizes. Lastly, most studies included in the meta-analysis were on coronary artery disease, with scarce data on heart failure. The type of cardiac disease can significantly influence the patient characteristics and functional capacity and the present review was unable to examine this in detail.

## Conclusions

This meta-analysis suggests that CR significantly enhances exercise capacity and cardiometabolic health across all BMI categories, showing no significant differences in results among obese, overweight, and normal-BMI individuals. Obese patients, although having lower baseline fitness levels, may demonstrate similar improvements in cardiorespiratory fitness and metabolic parameters, highlighting that excess BMI does not diminish the physiological advantages of CR. These findings underscore the necessity for universal accessibility to CR and advocate for its strong recommendation to all eligible patients, regardless of BMI. Future research must prioritize the standardization of CR intervention protocols and the incorporation of objective adherence metrics to provide better evidence.

## Data Availability

The original contributions presented in this study are included in the article/supplementary material, further inquiries can be directed to the corresponding author.

## Supplementary material

Supplementary Figure S1 Funnel plot to assess publication bias.

Supplementary Figure S2 Meta-analysis of change in BMI between obese vs non-obese groups undergoing CR.

Supplementary Figure S3 Meta-analysis of change in total cholesterol between obese vs non-obese groups undergoing CR.

Supplementary Figure S4 Meta-analysis of change in HDL between obese vs non-obese groups undergoing CR.

Supplementary Figure S5 Meta-analysis of change in triglycerides between obese vs non-obese groups undergoing CR.

Supplementary Figure S6 Meta-analysis of change in heart rate between obese vs non-obese groups undergoing CR.

Supplementary Figure S7 Meta-analysis of change in systolic blood pressure between obese vs non-obese groups undergoing CR.

Supplementary Figure S8 Meta-analysis of change in diastolic blood pressure between obese vs non-obese groups undergoing CR.
