# Supplementary figures and images for: Impact of body mass index on outcomes of cardiac rehabilitation: a systematic review and meta-analysis
Source: Front Cardiovasc Med. 2026 May 22;13:1757861. doi: 10.3389/fcvm.2026.1757861 (PMC13237748; doi:10.3389/fcvm.2026.1757861)

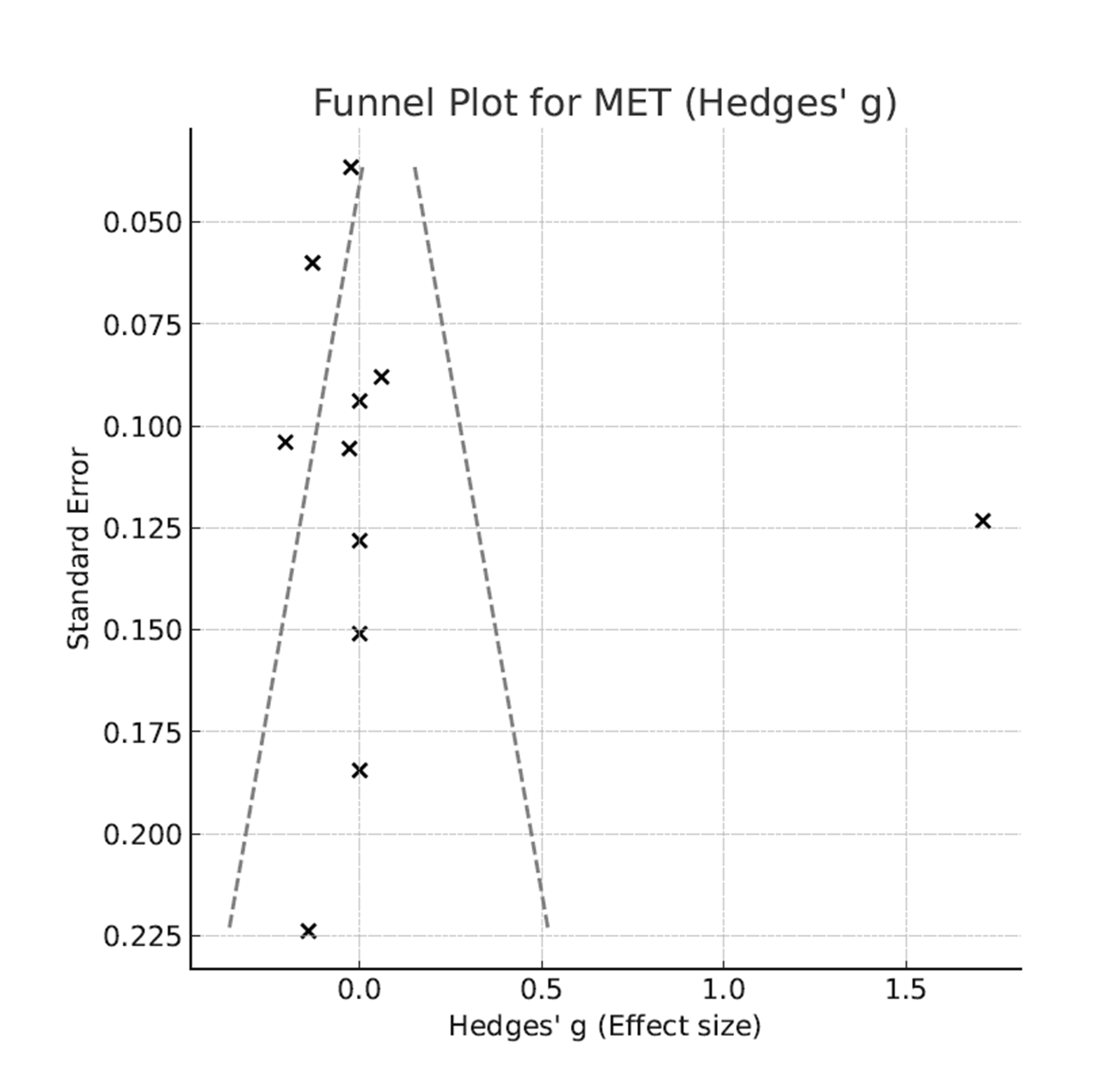

Supplement: Supplementary Figure S1 — Funnel plot to assess publication bias. [file Image1.tif]

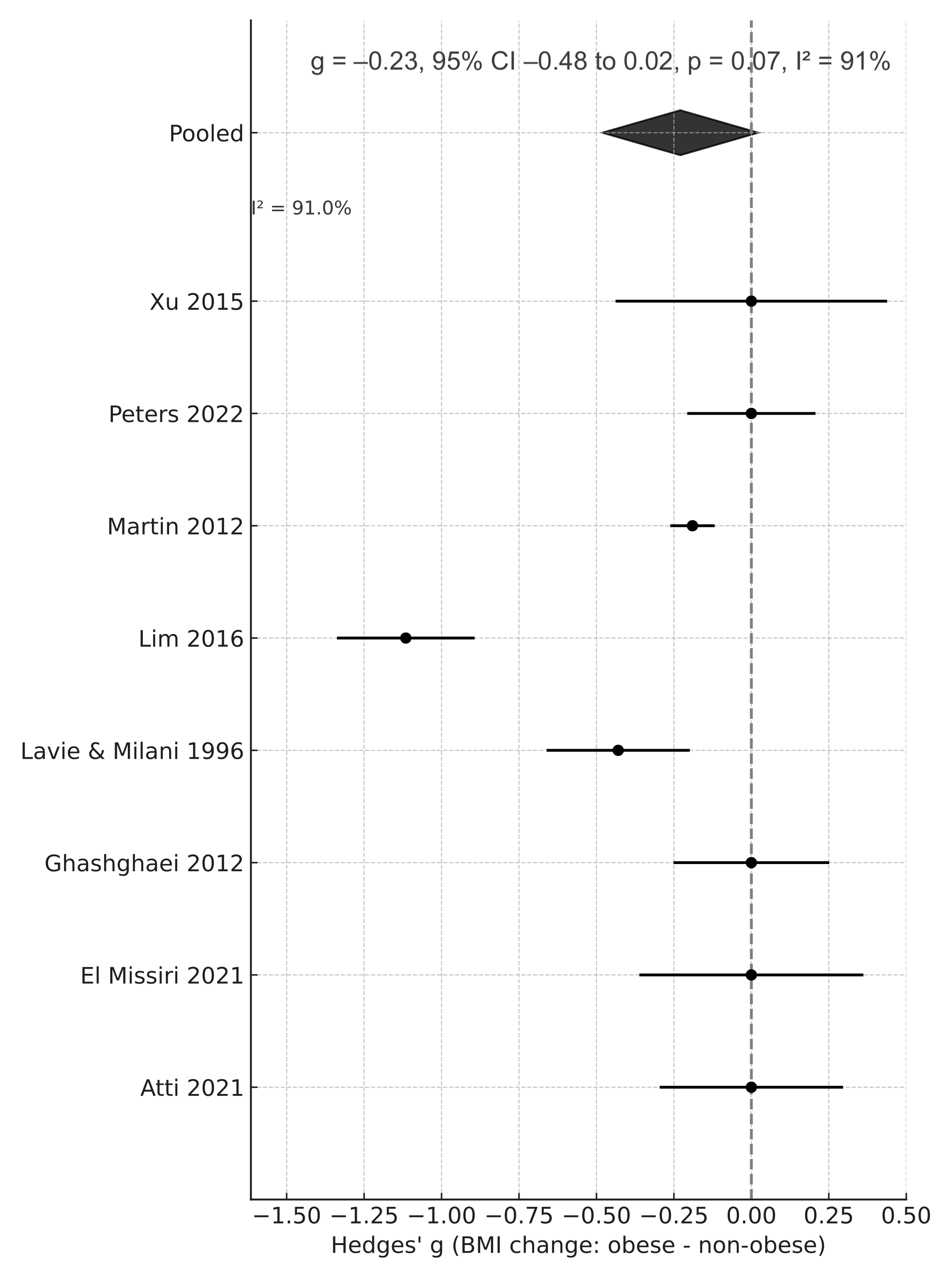

Supplement: Supplementary Figure S2 — Meta-analysis of change in BMI between obese vs non-obese groups undergoing CR. [file Image2.tiff]

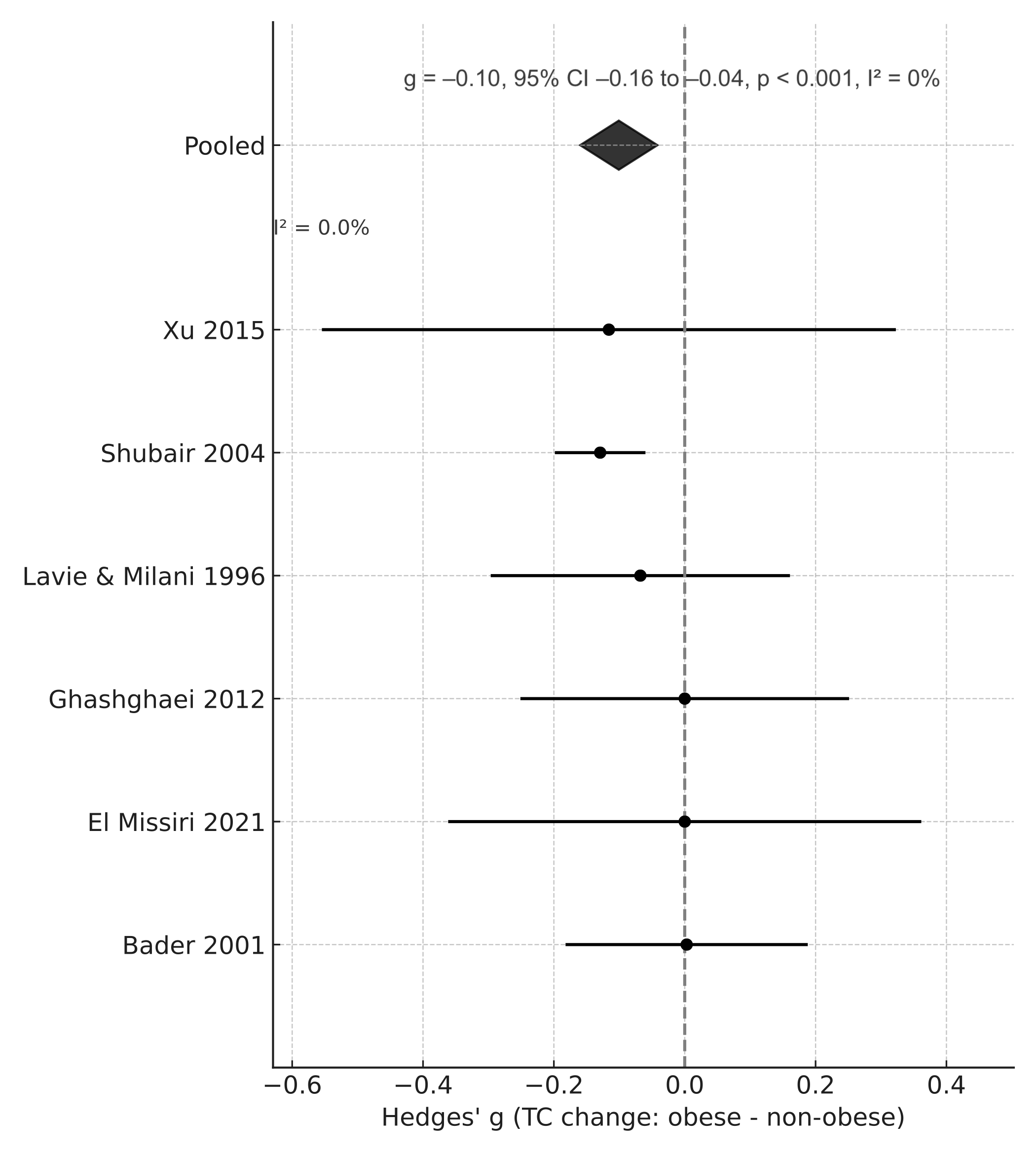

Supplement: Supplementary Figure S3 — Meta-analysis of change in total cholesterol between obese vs non-obese groups undergoing CR. [file Image3.tiff]

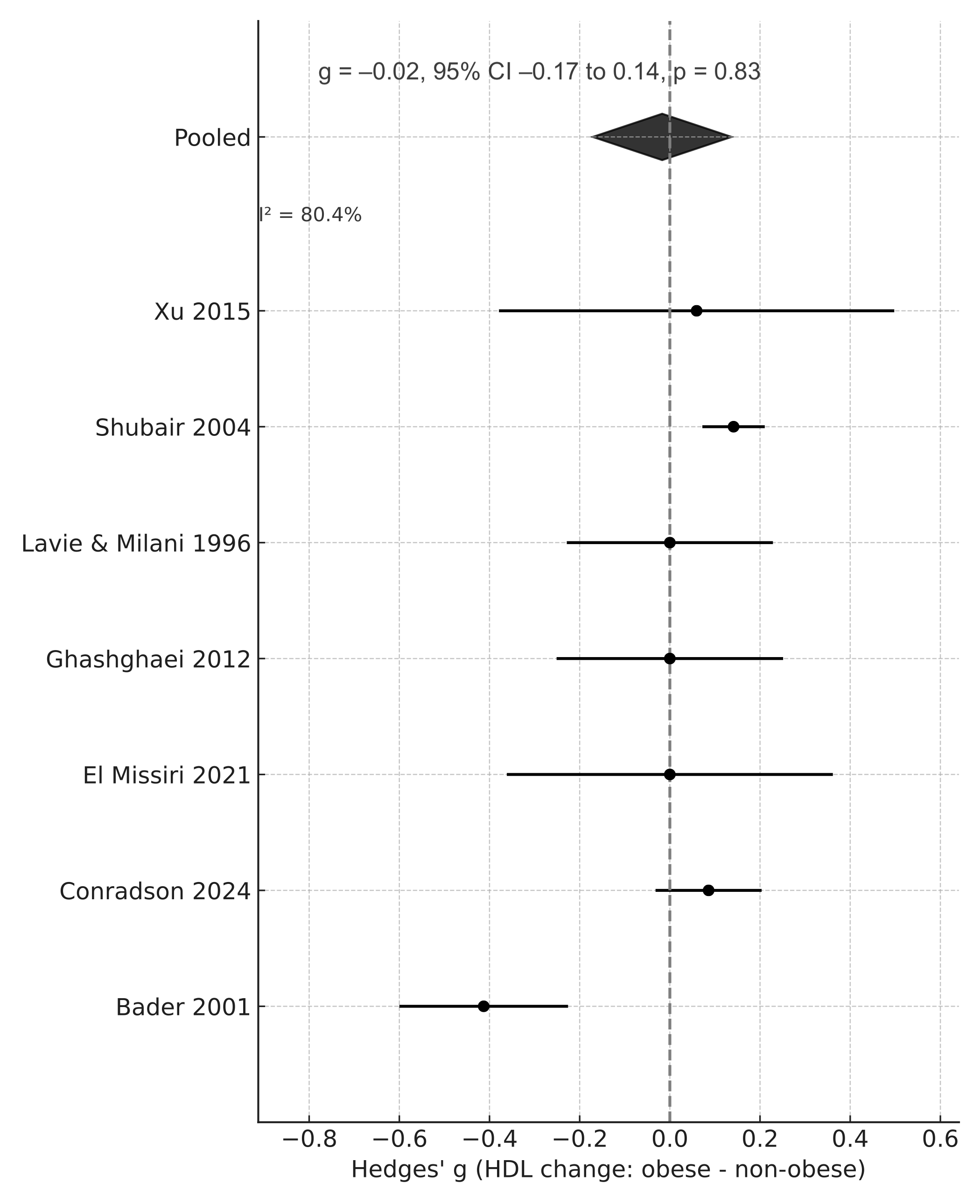

Supplement: Supplementary Figure S4 — Meta-analysis of change in HDL between obese vs non-obese groups undergoing CR. [file Image4.tiff]

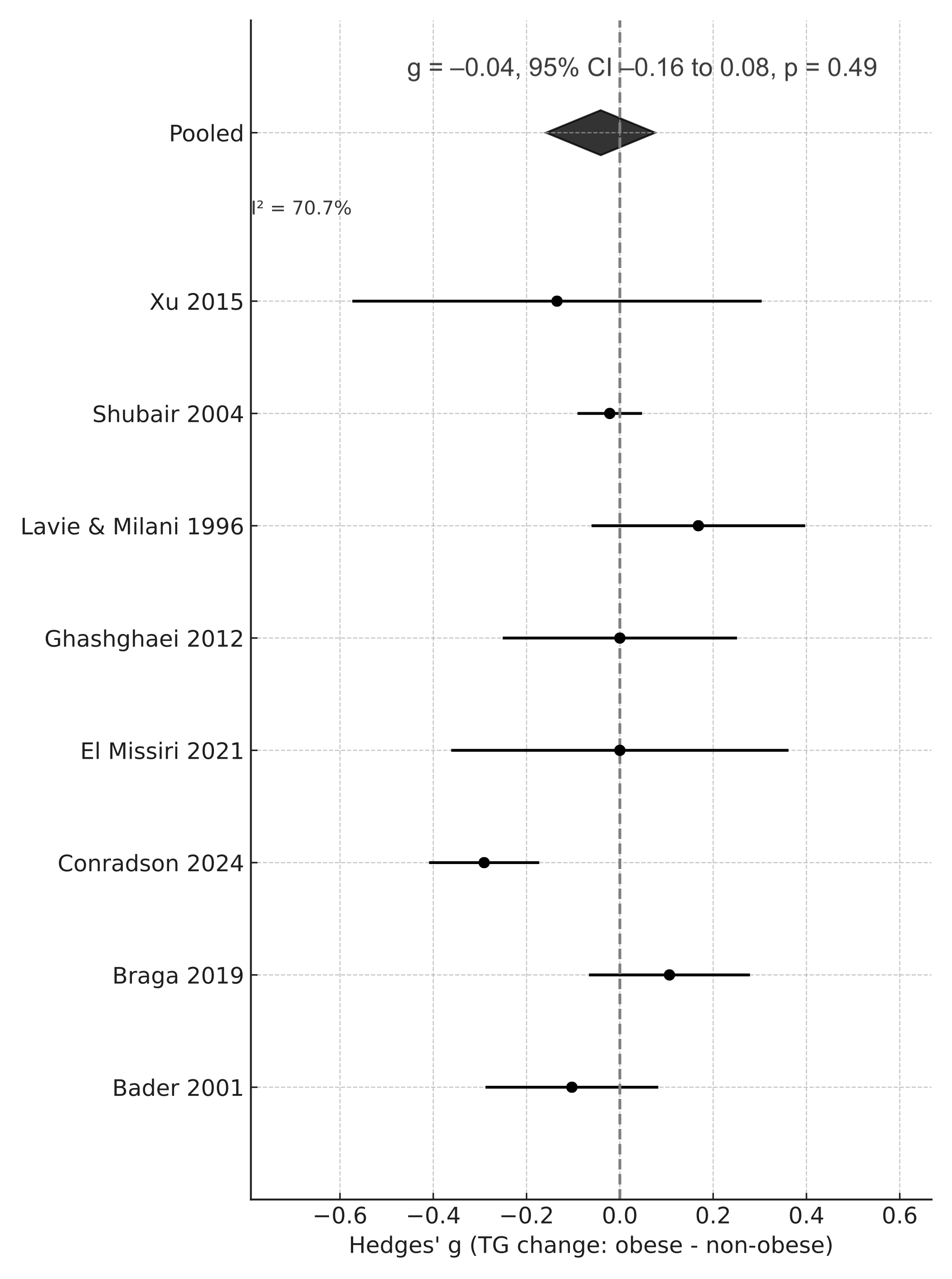

Supplement: Supplementary Figure S5 — Meta-analysis of change in triglycerides between obese vs non-obese groups undergoing CR. [file Image5.tiff]

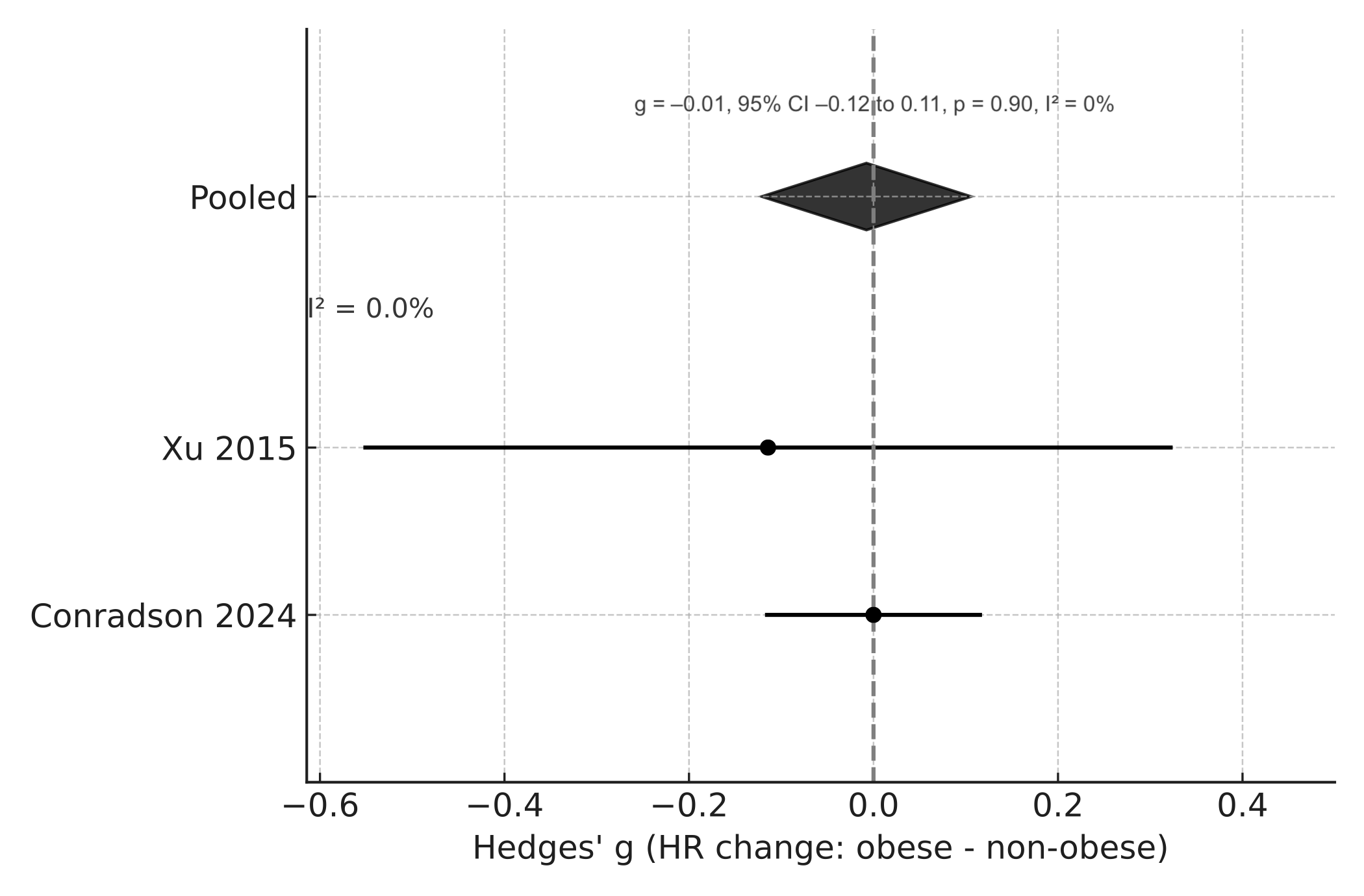

Supplement: Supplementary Figure S6 — Meta-analysis of change in heart rate between obese vs non-obese groups undergoing CR. [file Image6.tiff]

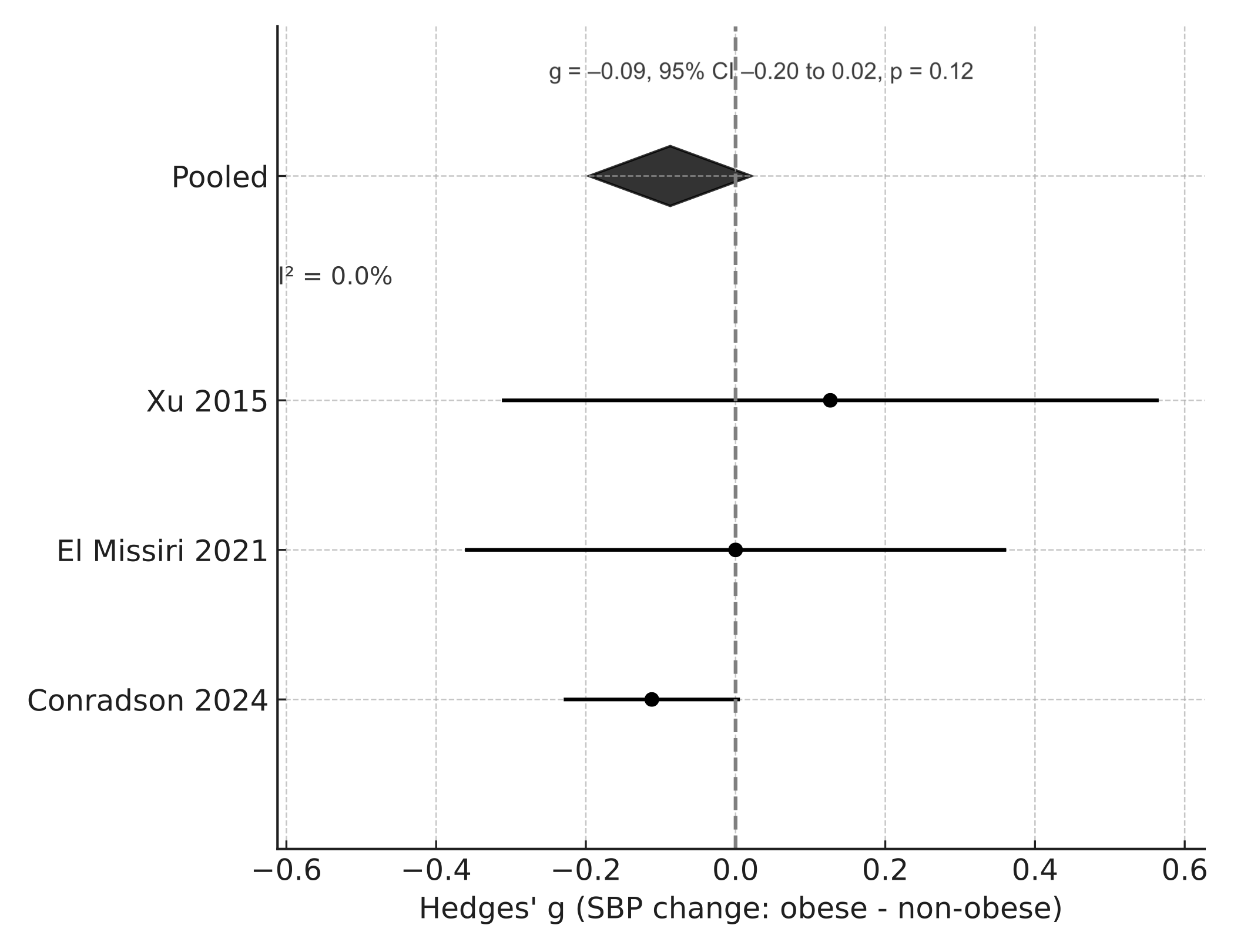

Supplement: Supplementary Figure S7 — Meta-analysis of change in systolic blood pressure between obese vs non-obese groups undergoing CR. [file Image7.tiff]

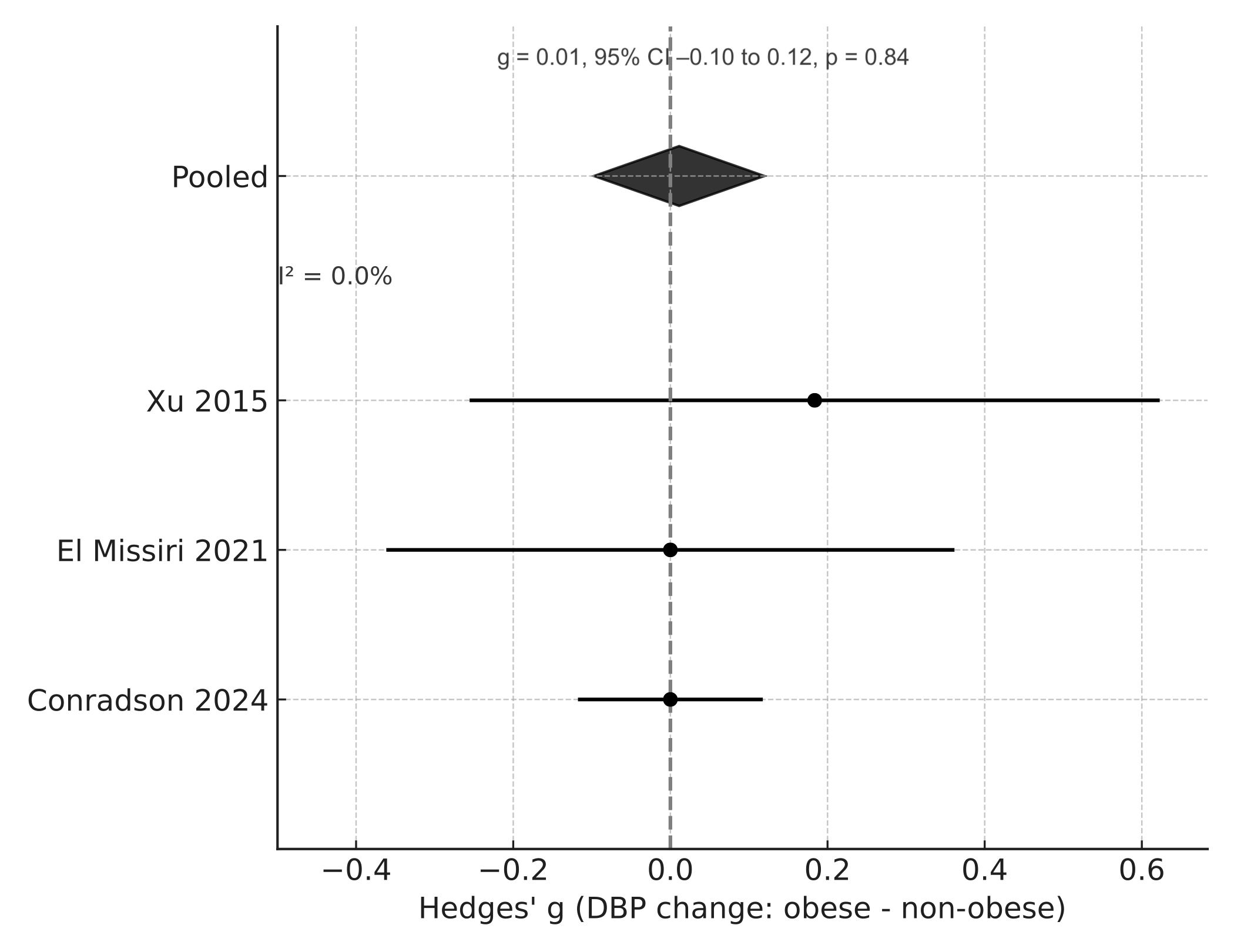

Supplement: Supplementary Figure S8 — Meta-analysis of change in diastolic blood pressure between obese vs non-obese groups undergoing CR. [file Image8.tiff]
