# Supplementary material for: Impact of body mass index on outcomes of cardiac rehabilitation: a systematic review and meta-analysis
Source: Front Cardiovasc Med. 2026 May 22;13:1757861. doi: 10.3389/fcvm.2026.1757861 (PMC13237748; doi:10.3389/fcvm.2026.1757861)
Supplement: Supplementary file 9 [file Table1.docx]

## Supplementary Table 1: Database Search Strategy

| Database | Search Terms / Strategy |
| --- | --- |
| PubMed (MEDLINE) | ("cardiac rehabilitation"[Mesh] OR "cardiac rehabilitation"[Title/Abstract] OR "exercise therapy"[Mesh] OR "exercise training"[Title/Abstract] OR "rehabilitation program*"[Title/Abstract] OR "secondary prevention"[Title/Abstract]) AND ("body mass index"[Mesh] OR "body mass index"[Title/Abstract] OR BMI[Title/Abstract] OR obesity[Title/Abstract] OR overweight[Title/Abstract] OR "body weight"[Mesh] OR "body composition"[Title/Abstract]) AND ("outcome*"[Title/Abstract] OR "functional capacity"[Title/Abstract] OR "exercise capacity"[Title/Abstract] OR "cardiorespiratory fitness"[Title/Abstract] OR "quality of life"[Mesh] OR "mortality"[Mesh] OR rehospitalization[Title/Abstract]) |
| Embase | ('cardiac rehabilitation'/exp OR 'cardiac rehabilitation':ti,ab OR 'exercise therapy'/exp OR 'exercise training':ti,ab OR 'secondary prevention':ti,ab) AND ('body mass index'/exp OR 'body mass index':ti,ab OR bmi:ti,ab OR obesity:ti,ab OR 'overweight'/exp OR 'overweight':ti,ab OR 'body composition'/exp OR 'body weight'/exp) AND ('treatment outcome'/exp OR outcome*:ti,ab OR 'functional capacity':ti,ab OR 'exercise capacity':ti,ab OR 'cardiorespiratory fitness':ti,ab OR 'quality of life'/exp OR mortality:ti,ab OR rehospitalization:ti,ab) |
| Web of Science | TS = ("cardiac rehabilitation" OR "exercise therapy" OR "exercise training" OR "secondary prevention") AND TS = ("body mass index" OR BMI OR obes* OR overweight OR "body composition" OR "body weight") AND TS = ("outcome*" OR "functional capacity" OR "exercise capacity" OR "cardiorespiratory fitness" OR "quality of life" OR mortality OR rehospitalization) |
| Scopus | (TITLE-ABS-KEY("cardiac rehabilitation" OR "exercise therapy" OR "exercise training" OR "secondary prevention")) AND (TITLE-ABS-KEY("body mass index" OR BMI OR obesity OR overweight OR "body composition" OR "body weight")) AND (TITLE-ABS-KEY(outcome* OR "functional capacity" OR "exercise capacity" OR "cardiorespiratory fitness" OR "quality of life" OR mortality OR rehospitalization)) |
