# Supplementary material for: Impact of body mass index on outcomes of cardiac rehabilitation: a systematic review and meta-analysis
Source: Front Cardiovasc Med. 2026 May 22;13:1757861. doi: 10.3389/fcvm.2026.1757861 (PMC13237748; doi:10.3389/fcvm.2026.1757861)
Supplement: Supplementary file 10 [file Table2.docx]

# Supplementary Table 2: Sensitivity Analysis for Change in MET

| Excluded Study | Hedges' g | 95% CI | I² (%) |
| --- | --- | --- | --- |
| Atti 2021 | 0.046 | (-0.239, 0.331) | 96.0 |
| Braga 2019 | 0.041 | (-0.257, 0.339) | 96.0 |
| Conradson 2024 | 0.061 | (-0.260, 0.381) | 95.9 |
| El Missiri 2021 | 0.061 | (-0.221, 0.343) | 96.0 |
| Ghashghaei 2012 | 0.037 | (-0.249, 0.322) | 96.0 |
| Gunstad 2007 | 0.068 | (-0.223, 0.359) | 95.9 |
| Lavie & Milani 1996 | 0.077 | (-0.209, 0.363) | 95.9 |
| Lim 2016 | -0.116 | (-0.202, -0.030) | 47.9 |
| Martin 2012 | 0.054 | (-0.298, 0.407) | 95.9 |
| Mittal 2024 | 0.091 | (-0.189, 0.372) | 95.8 |
| Xu 2015 | 0.059 | (-0.221, 0.338) | 96.0 |

CI, confidence intervals
