# Supplementary material for: Impact of body mass index on outcomes of cardiac rehabilitation: a systematic review and meta-analysis
Source: Front Cardiovasc Med. 2026 May 22;13:1757861. doi: 10.3389/fcvm.2026.1757861 (PMC13237748; doi:10.3389/fcvm.2026.1757861)
Supplement: Supplementary file 11 [file Table3.docx]

Supplementary Table 3: Risk of bias analysis

| Author | Selection | Comparability | Outcomes | Total score |
| --- | --- | --- | --- | --- |
| Lavie et al. | 4 | - | 2 | 6 |
| Bader et al. | 4 | - | 2 | 6 |
| Shubair et al. | 4 | - | 2 | 6 |
| Sierra-Johnson et al. | 4 | - | 3 | 7 |
| Gunstad J et al. | 4 | - | 2 | 6 |
| Ghashghaei et al. | 4 | - | 2 | 6 |
| Martin et al. | 4 | - | 3 | 7 |
| Xu et al. | 4 | - | 2 | 6 |
| Lim et al. | 4 | - | 2 | 6 |
| Pieters et al. | 4 | - | 2 | 6 |
| Braga et al. | 4 | - | 2 | 6 |
| Terada et al. | 4 | - | 2 | 6 |
| Atti et al. | 4 | - | 2 | 6 |
| El Missiri et al. | 4 | - | 2 | 6 |
| Khan et al. | 4 | - | 3 | 7 |
| Peters et al. | 4 | - | 2 | 6 |
| Conradson et al. | 4 | - | 2 | 6 |
| Mittal et al. | 4 | - | 2 | 6 |
